# Wanted: A vocabulary for talking about involuntary behaviors associated with Lesch‐Nyhan disease

**DOI:** 10.1002/jmd2.12210

**Published:** 2021-03-23

**Authors:** Kenneth L. Robey, Daniel C. Balboni

**Affiliations:** ^1^ Quality Improvement and Patient Safety Matheny Medical and Educational Center Peapack New Jersey USA; ^2^ Psychological Services Matheny Medical and Educational Center Peapack New Jersey USA

**Keywords:** aggression, intent, Lesch‐Nyhan, self‐injury, volition

## Abstract

Lesch‐Nyhan disease (LND) is a rare genetic disorder with an unusual behavioral phenotype that includes severe and involuntary self‐injury requiring the near constant use of protective devices and, for some individuals, dental extraction. Often, the person with LND also engages in emotional self‐injury in the form of both self‐sabotage and behaviors directed toward others that will have a negative social consequence. When these self‐destructive behaviors present themselves, it is sometimes challenging for caregivers, professionals, or other observers to fully recognize their lack of volition. It is an even greater challenge to accurately and convincingly convey their involuntary nature to medical students, colleagues, school staff, or even family members who might be unfamiliar with the disorder. It is difficult to find words to clearly and adequately convey the essence of behaviors like those that we find in LND without, in some way, implying intent.

**Synopsis**

It is challenging to describe the involuntary behaviors associated with Lesch‐Nyhan disease without inadvertently implying intent.

“Concupiscence and force are the source of all our actions. Concupiscence causes voluntary, force involuntary actions.”

Blaise Pascal (1623‐1662) ^1^

In a passage of *Pensées* published posthumously in 1670, Pascal^1^ divided actions into two categories, voluntary and involuntary. He believed that concupiscence (ie, ardent desire or longing) is the motivational driver of our voluntary actions, and external force (ie, coercion) is the driver of our involuntary actions. What Pascal's dichotomy does not accommodate, however, are actions that originate organically within the individual yet are outside the control of, and often against the desires of, the individual. They might even be behaviors that produce severe physical or emotional self‐harm. Such are the actions that characterize the behavioral phenotype of Lesch‐Nyhan disease (LND).

LND is a genetic (X‐linked recessive) disorder of purine metabolism occurring in roughly one in 380 000 live births.^2^ It involves the near absence of the enzyme hypoxanthine guanine phosphoribosyltransferase. Persons with classic LND typically have renal dysfunction, dystonia, dysarthric speech, and varying degrees of cognitive impairment ranging from profound deficit to near normal functioning.^3^ The behavioral hallmark symptom of LND is severe and involuntary self‐injury.

The individual with LND might engage in self‐biting of fingers or lips, possibly to amputation, eye‐poking, scratching, head‐banging, or other self‐injuries.^4^, ^5^ Individuals with LND typically use and appreciate a range of protective devices such as wrist cuffs, arm splints, night time tie downs, and bed bumpers.^4^, ^6^ Dental extraction to prevent injury due to biting has become a mainstay of treatment among those for whom biting is a pervasive problem.^7^

Persons with LND do not want to hurt themselves. If their protective devices are removed or not properly applied, they will be anxious, and if their hands become free they might be terrified of the damage they might cause by poking their eyes or biting their fingers. At times, they are their own best advocates, telling caregivers when their wrist cuffs are too loose, or even when they are beginning to fray and need to be replaced.

Beyond the physical self‐injury, the behavioral phenotype might also include behaviors that are directed toward others.^5^, ^8^ These outwardly directed behaviors might involve such physical actions as hitting, kicking, biting, head‐butting, or hair‐pulling. They might take the form of verbal behaviors, including profanities, ethnic slurs, insults, or inappropriate sexual comments. The more complex of these verbal behaviors might take the form of false accusations of abuse by service providers or other forms of social manipulation. Like the physical self‐injury, these behaviors directed toward others are thought to serve a self‐injurious function—an “emotional self‐injury” or “psychosocial self‐injury” of sorts.^5^ Individuals with LND often do things that bring a negative emotional or psychosocial consequence on themselves; things that will cause others to view them and potentially treat them negatively.

Caregivers also report instances of “self‐sabotage” in which the individual might deny himself/herself an activity that he or she enjoys. The person with LND might be excited in the days leading up to a trip to a favorite restaurant or sports event, then ask to stay back when the time comes to leave for the event. Or the individual might devote considerable time and energy to a project, perhaps composing a poem or painting, then act to destroy their work.

When queried about their behaviors, particularly about the cause and nature of their behaviors, we have found people with LND to often be at a loss. They do not describe the behaviors as intentional or as instrumental. Nor do they typically describe them as being cathartic in nature; as involving some release of pent up energies. Rather, their explanations are more existential. “This is just what my body does. I can't stop it.”

When teaching medical students or other professionals‐in‐training about LND, or when counseling family members, caregivers, or school staff, we often find ourselves comfortable conveying what we know about the incidence of the disorder. We are equally comfortable talking about the extent of physical self‐injury that we see in these individuals. We are comfortable talking about the psychological, medical, and technological strategies that are thought to be the most effective in addressing the behaviors. Where we stumble, however, is in finding words to convey the *nature* of the self‐injurious behaviors. When telling students about things that persons with LND do to other people, such as when patients kick caregivers who are trying to put socks on their feet; when they accuse a long‐time and appreciated aide of abusing them; or when they draw visitors close by whispering, then head‐butt them or spit in their faces as they lean in, our descriptions are particularly awkward.

At times individuals with LND will warn their caregiver that a behavior is coming, perhaps telling the caregiver to move because they are going to spit on them or asking someone to step back so that they cannot be kicked. The individual might tell a visitor who does not know them, “I might say some things but I won't really mean them.” Not only do they show remorse for a behavior after the fact, but there is a desire to prevent a behavior from impacting another.

Our awkwardness in talking about LND behaviors is, in part, because we do not fully understand the physiological bases of the behaviors. In part, it is because we have difficulty relating to a situation in which one is behaving in a way that so directly hurts themselves or people that they might care about. It is also in part because we simply have not found the appropriate *vocabulary* to describe the behaviors.

Labels that are applied to the behaviors associated with LND include “self‐harm,” “self‐injury,” and “self‐mutilation.” In the American Psychological Association *Dictionary of Psychology*, the definitions of self‐harm, self‐injury, and self‐mutilation all reference the definition of “deliberate self‐harm (DSH)”.^9^ The definition of DSH is “the intentional, direct destruction of body tissue (most commonly by cutting, burning, scratching, self‐hitting, self‐biting, and head banging) without conscious suicidal intent but resulting in injury severe enough for tissue damage to occur.” Based on this definition, terms like self‐harm, self‐injury, and self‐mutilation are clearly associated with intentional action to cause harm to one's self.

Sometimes, even professionals very familiar with LND will use a word implying intent to convey that an event related to an LND behavior was not an accident. In a recent safety event report submitted by a staff person at our facility, it was reported, “Patient *deliberately* hit forehead on bathroom door frame when exiting shower.” This reporter might have had a clear understanding of the involuntary nature of the behavior she observed, but chose the adverb “deliberately” as a simple and economical way to convey that the incident was not an accident; rather the patient was compelled (presumably without intent) to hit his head against the door frame.

Contributing to the fuzzy relationship between the concept of involuntariness and the available descriptors is recognition that LND behaviors do seem to serve an instrumental purpose (as described earlier, they are thought to be physiologically energized actions serving the purpose of physical or emotional self‐injury). When one performs an action that serves a purpose, we generally assume the action to be intentional. As such, words that convey instrumentality typically also convey intent. Verbs like “try” and “aim,” for example, connote instrumentality and they also imply intent.

The most common descriptor we use in discussing those LND behaviors that are directed toward others is “aggressive.”^4^, ^10^, ^11^ While Buss^12^ held a rather simple view of aggression as any behavior that harms another, dominant social psychological definitions of aggression specify that there is intent to harm and not simply the delivery of harm.^13^, ^14^, ^15^ The application of the adjective “aggressive” to LND behaviors, based on these definitions, is problematic.

At a surface level, the word “aggressive” does seem to make sense when watching LND behavior. When we observe an individual with LND who is being bathed by a caregiver swing his arm to hit the caregiver as his protective straps are released, the word “aggressive” quite readily comes to mind. But when that young man apologizes genuinely and profusely for his action only to find himself again swinging his arm at the caregiver, the word “aggressive” no longer fully resonates. The surprise often experienced by people with LND when they act toward another, along with the minimal latency with which the behaviors occur, suggests lack of intention prompting Bozano et al^8^ to find the word “aggression” to be an inappropriate way to describe at least some LND behaviors.

We have heard caregivers, physicians, and families use other words to convey the lack of intent present in LND behavior as well, but those words too are lacking. Words like “unintentional” or “accidental” convey a lack of awareness of consequence, which we do not hear reported by individuals diagnosed with LND. They are well aware of the consequences of their behaviors. Language associating LND behaviors with compulsion, when used in a psychiatric context, also misses the mark. Compulsions, according to ICD‐11, are behaviors or mental acts “that the individual feels driven to perform in response to an obsession, according to rigid rules, or to achieve a sense of ‘completeness’.”^16^ Individuals with LND do not describe their behaviors as being associated with conscious thoughts, let alone obsession, and the behaviors certainly are not associated with rigid rules or with a sense of completeness.

The words “habitual” or “addictive” also fall short. Habitual implies the behavior is shaped, often with purpose, by the individual, another person, or the environment. Addictive behaviors, as defined in the American Psychological Association *Dictionary of Psychology*, are “actions, often obsessive and destructive, that are related to one's abuse of or dependence on a substance that dominates one's life.”^9^ The dictionary acknowledges the term addict is also used “colloquially to refer to a person with compulsive behavior such as persistent gambling.”

The word “unvoluntary” has been suggested as a descriptor for tic behaviors, such as those associated with Tourette syndrome, that seem to fall in a grey area between voluntary and involuntary. In that grey area, an urge to move precedes the movement and the movement can be suppressed for a period of time.^17^, ^18^ Key to this idea is that there is a premonitory urge that precedes the movement. There is conceptual value to creating a semantic space between voluntary and involuntary and, to the extent that people with LND behaviors might at times be able to suppress a behavior for a short period of time, those behaviors might appropriately fall into that space. Anecdotally, patients have told us that they have some sense of when they are likely to self‐injure or act against another person, but it is unclear whether those instances involve premonitory urges or a recognition of a trigger, such as a caregiver within a range in which he or she can be struck or spit upon. Regardless, stating that a set of behaviors is “unvoluntary” does little beyond the use of the word “involuntary” to facilitate discussion of the behaviors with healthcare students, caregivers, school teachers, or others.

Complicating things further, the initiation of LND behaviors that are directed toward others is not necessarily random and their enactment might, at times, involve some degree of *selection or targeting* (themselves words that imply intent and, in the case of the latter, perhaps malice). We have observed LND behaviors, often taking the form of accusations of abuse, directed toward caregivers with whom the individual with LND feels unsafe because the caregiver is inexperienced or does not fully understand proper management of LND behaviors. Conversely, we have also seen individuals whose behaviors seem to be directed most frequently to those with whom they have the greatest emotional attachment, such as parents or trusted caregivers. Hurting someone who you care about and who cares about you will produce the greatest emotional self‐injury. It is difficult to fathom, let alone explain to others, how this selectivity or targeting might occur in the absence of intent.

When describing LND behaviors to students or others who are unfamiliar with LND, it does help somewhat at the outset to refer to the behaviors, both those directed toward the self and those directed toward others, as having a reflexive quality or as being “tic‐like” in their initiation. But referring to one's behavior as “tic‐like” does not adequately convey the individual's struggle as they both experience the behavior and, at the same time, fight its enactment. Individuals with LND have told us that the impulse to act on a negative behavior and the thought to fight the release of the behavior occur simultaneously. With each behavior, the individual struggles between its enactment and attempts to curb its release.

Consider the case of an individual diagnosed with LND who says that he has pain and, as an LND behavior, involuntarily portrays that pain as considerably worse than it is. This individual frequently meets with medical students to speak with them about the experience of living with the diagnosis and shares an example with them to demonstrate the danger of the involuntary behavior. He was recovering in the hospital after a surgery and, in his words, “lied about” his pain, stating it was significantly worse than it was. This individual, as a result, suffered complications due to being over‐medicated. This was, by his account, the potential outcome that spawned the behavior. He was aware that his behavior put him in danger, yet he was not for a moment consciously motivated to self‐harm. In this example, simply calling his fabricated complaint an involuntary tic‐like behavior does not fully capture the event or his internal struggle.

Some caregivers have expressed to us that they have observed projectile vomiting toward a caregiver or visitor, a behavior not normally within one's conscious control, presumably as a form of social/emotional self‐injury (the first author has found himself jokingly telling medical students that the projectile vomiting is often with “deadly accuracy,” inadvertently implying not only conscious activity but also skill on the part of the individual with LND). Even those behaviors that are outside the individual's conscious control can be challenging to describe without implying intent.

When professionals in the mental health field discuss “self‐harm behaviors” or “self‐sabotage,” it is sometimes paired with a discussion of “secondary gain.” This is usually the emotional, monetary, social, or chemical addiction‐based gain which motivated the self‐harm.^19^, ^20^ The assumption here is that a person would not harm themselves intentionally (or perhaps in the case of LND, unintentionally) without receiving something valued in return. For example, a person may drink to a point where they damage their body severely, but are self‐medicating to quiet intrusive thoughts or memories beyond their ability or willingness to process. Rather than secondary gain, individuals with LND might experience what one could think of as “secondary harms.” They experience the self‐harm of the behavior directly, and paired with that behavior might be secondary harms such as: the loss of a relationship, loss of trust, or loss of an activity they were enjoying. Consider one individual with LND who loves the New York Yankees and is scheduled to go on a trip to see a game. On the day of the game, he states he feels sick and is in excruciating discomfort and ends up not going to the game. This individual not only misses the game, but also spends the entire day in bed, receiving medication he does not need, misses day program activities and is limited in social interactions. This individual can then discuss this involuntary self‐sabotage as well as all of the losses that accompanied it, stating that he had not experienced any of the symptoms he claimed and that while he was making the claims he was hoping that they would be ignored. By illustrating what might be layers of undesirable outcomes of LND behaviors, perhaps this notion of secondary harm can help to convey lack of intent.

We suppose that, to some extent, the same dilemma in trying to convey the involuntary nature of LND behaviors applies to those behaviors and behavioral tendencies that are initiated and/or energized through other unconscious processes, such as implicit bias, the unconscious attribution of characteristics, or qualities to members of a social outgroup.^21^ Or perhaps there is a similar awkwardness in describing behaviors associated with psychosis. In the case of the former, however, one might argue that behaviors associated with implicit bias are not completely contrary to the individual's wishes as they do represent the individual's leanings or belief system if even at an unconscious level. In the case of the latter, while the individual might not in their latent state wish to display or experience psychotic behaviors, within the context of the psychotic episode we might think of the behaviors as being consistent with and motivated by the individual's internal experience at that point in time.

To varying degrees, behaviors associated with LND, with psychosis, with implicit attitudes, and even with habits or compulsions, all involve limits to (or perhaps deficits in) one's self‐agency. Each defies clear phenomenal description of the degree to which they represent willful behavior. In any event, the behaviors associated with LND are unique in their contrariness to the individual's deepest wishes to remain safe, both at the instant in which the behaviors occur and across the broader arc of the individual's experience.

Where language seems to fail us most in teaching health care students, professionals, caregivers, and others about LND behaviors is in the paucity of verbs or verb phrases to denote involuntary action. We find ourselves saying that a particular person with LND “*tries* to bend his fingers back,” or that another “*favors* those self‐injurious behaviors that involve an external surface, like rubbing his ears on the wheelchair headrest.” In telling students about behaviors directed toward others, we lapse into statements like, “the behaviors *are often targeted* toward staff who care for these individuals,” or “the individual might *attempt* to pull someone's hair,” or “the verbal behaviors *are sometimes meant* to shock or upset others.” To varying degrees, all of these verbs or verb phrases imply intent.

Having posed the question of how to appropriately describe Lesch‐Nyhan behaviors to those who are unfamiliar with the disorder, and having devoted the bulk of this paper to an exploration of the shortcomings of available descriptors, we are obliged to offer some suggestions. The above analysis illustrates a particular need for a means to convey the internal struggle experienced by people with LND as they try to curb their own largely unchecked behaviors. Notwithstanding our earlier observation that the psychiatric use of the term “compulsion” does not accurately describe LND behaviors, the popular use of the verb form, “compel” does have a better fit. It is helpful to think of the individual as internally “compelled” to self‐injure, with the behavior initiated and driven by an internal spontaneous incitement. We have also thought of LND behaviors as “inexorable,” resistant to internal persuasion, prohibition, or inhibition. Together, perhaps the verb “compelled” and adjective “inexorable” help to illustrate the internal struggle that individuals with LND have conveyed to us that they experience.

Any concept that manifests outside our common experience is inherently difficult to articulate. Our difficulties in finding appropriate words to describe LND behaviors, perhaps thankfully, reflect the relative rarity with which we see the scope, intensity, and complexity of the kinds of behaviors shown by those who have the disorder.

## CONFLICT OF INTEREST

The authors declare no potential conflict of interest.

## AUTHOR CONTRIBUTIONS

Kenneth L. Robey was involved in the conceptualization, drafting and editing of this paper. Daniel C. Balboni was involved in the conceptualization of portions of this paper and in the drafting and editing of the paper.

## ETHICS STATEMENT

This work did not contain any studies with human or animal subjects conducted by either Kenneth L. Robey or Daniel C. Balboni.

## PATIENT CONSENT STATEMENT

Not applicable.

## Data Availability

Data sharing is not applicable to this article as no new data were created or analyzed in this study.
